# Supplementary material for: A global meta-analysis of soil organic carbon in the Anthropocene
Source: Nat Commun. 2023 Jun 22;14:3700. doi: 10.1038/s41467-023-39338-z (PMC10287672; doi:10.1038/s41467-023-39338-z)

Supplementary Materials to **A Global Meta-analysis of Soil Organic Carbon in the Anthropocene**

Damien Beillouin[1,2*], Marc Corbeels[3,4], Julien Demenois[3,5,6], David Berre[3,7,8], Annie Boyer[9], Abigail Fallot[10,11], Frédéric Feder[12,13], Rémi Cardinael[3,14,15]

1. CIRAD, UPR HortSys, F-34398 Montpellier, France
2. HortSys, Univ Montpellier, CIRAD, Montpellier, France
3. AIDA, Univ Montpellier, CIRAD, Montpellier, France
4. IITA, Nairobi, Kenya
5. CIRAD, UPR AIDA, Turrialba 30501, Costa Rica
6. CATIE, Centro Agronómico Tropical de Investigación y Enseñanza, Turrialba 30501, Costa Rica
7. CIRAD, UPR AIDA, Bobo-Dioulasso, Burkina Faso
8. CIRDES, USPAE, Bobo-Dioulasso, Burkina Faso
9. CIRAD, DGDRS, DIST, F-34398 Montpellier, France
10. CIRAD, UMR SENS, Montpellier, France
11. SENS, Univ Montpellier, CIRAD, Montpellier, France
12. CIRAD, UPR Recyclage et Risque, Montpellier, France
13. Recyclage et Risque, Univ Montpellier, CIRAD, Montpellier, France
14. CIRAD, UPR AIDA, Harare, Zimbabwe
15. Department of Plant Production Sciences and Technologies, University of Zimbabwe, Harare, Zimbabwe

* corresponding author

* *damien.beillouin@cirad.fr*

Supplementary Figure 1. Categorization of available meta-analyses according to the metric used to calculate the effect-size, the role of the SOC variable and the type of carbon analysed. The total number of meta-analyses with main effect on SOC is 230 (202+33 – 5 meta-analyses presenting both effect-sizes as ratio and other effect sizes).

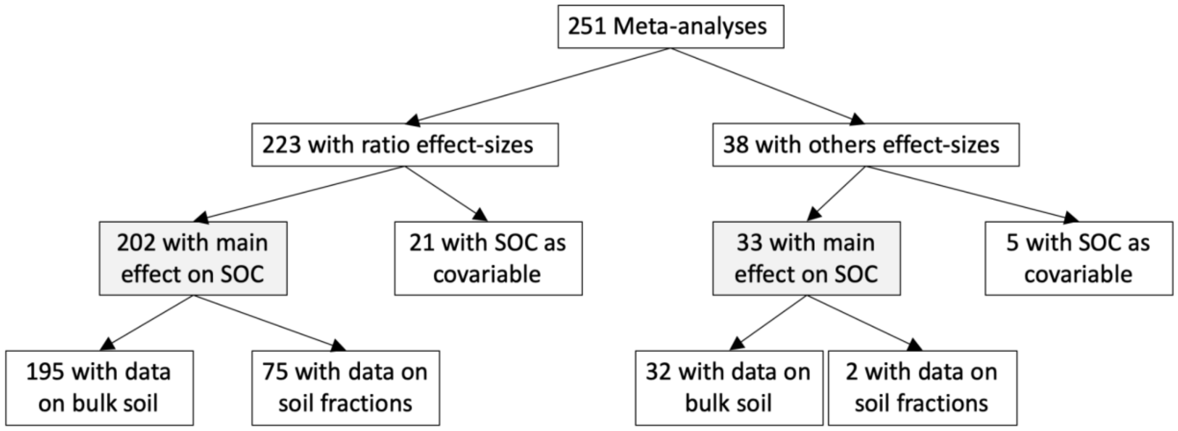

The list and characteristics of the meta-analyses is available here: https://rpubs.com/dbeillouin/List_studies

Supplementary Figure 2. Comparisons of the results between frequentist and Bayesian inference for land-use change (a.), Management (b) and Climate change (c) factors. Best frequentist and Bayesian models were selected based on their AIC and WAIC, respectively. Each point represents a mean calculated effect-size. Colors indicate the difference between the two models.

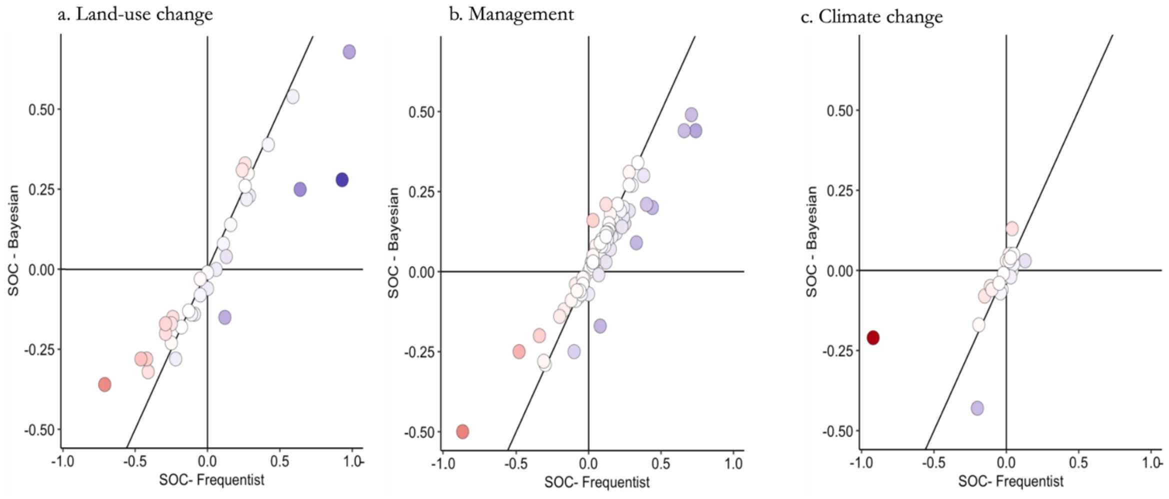

Supplementary Figure 3. Comparisons of the results between the best frequentist mixed model (selected based on their AIC) and fixed effect model for land-use change (a.), Management (b) and Climate change (c) factors. Each point represents a mean calculated effect-size. Colors indicate the difference between the two models. Details of the information used to make this graph are available at : https://rpubs.com/dbeillouin/Suppl_soil_depth

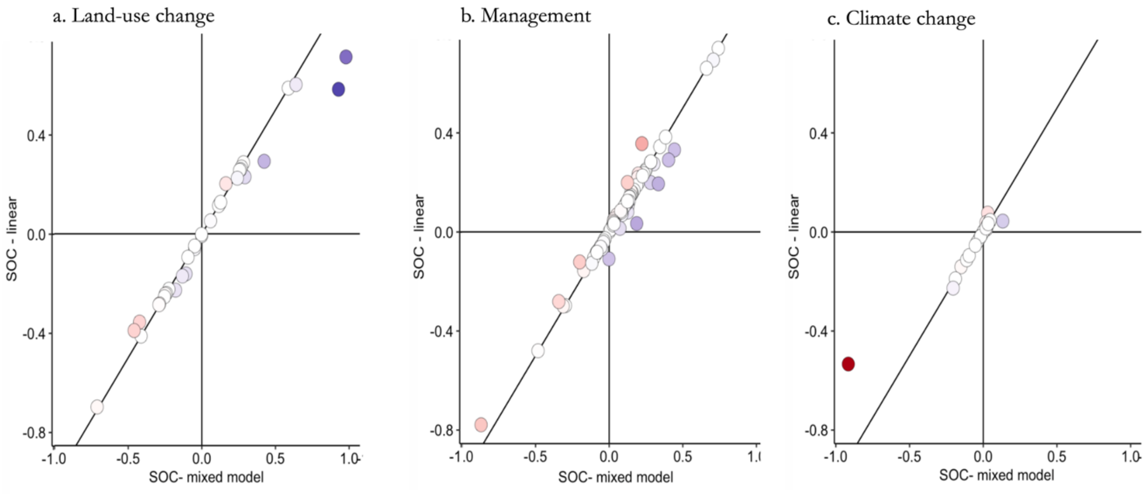

Supplementary Figure 4. Funnel plot: Mean effect size vs. their precision for the whole database. Each point represents an effect-size. The bubble size is proportional to the number of Data. Color represent the various land-use type.

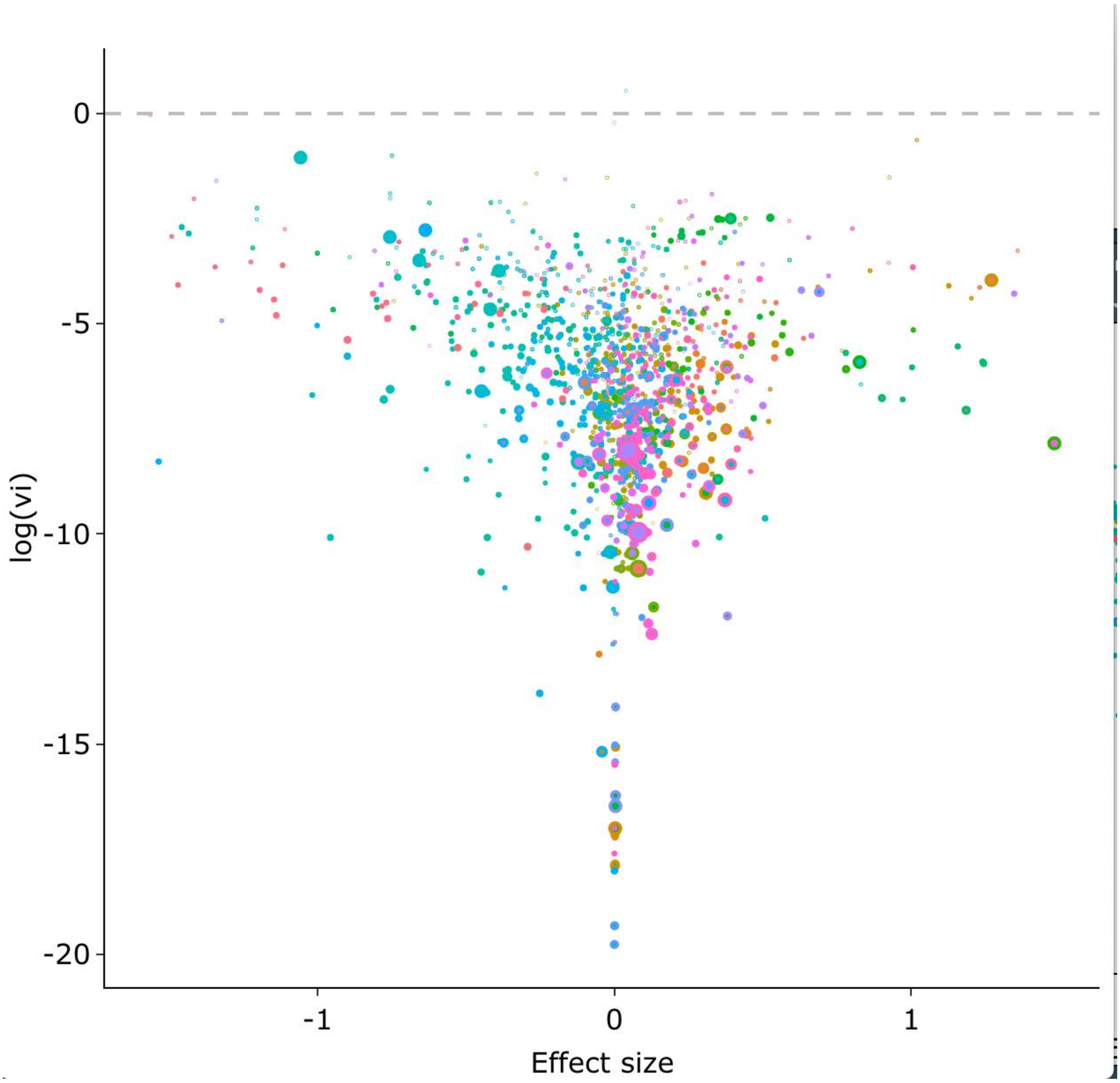

Supplementary Figure 5. Comparison between linear and trim and fill method impact on the mean estimates.

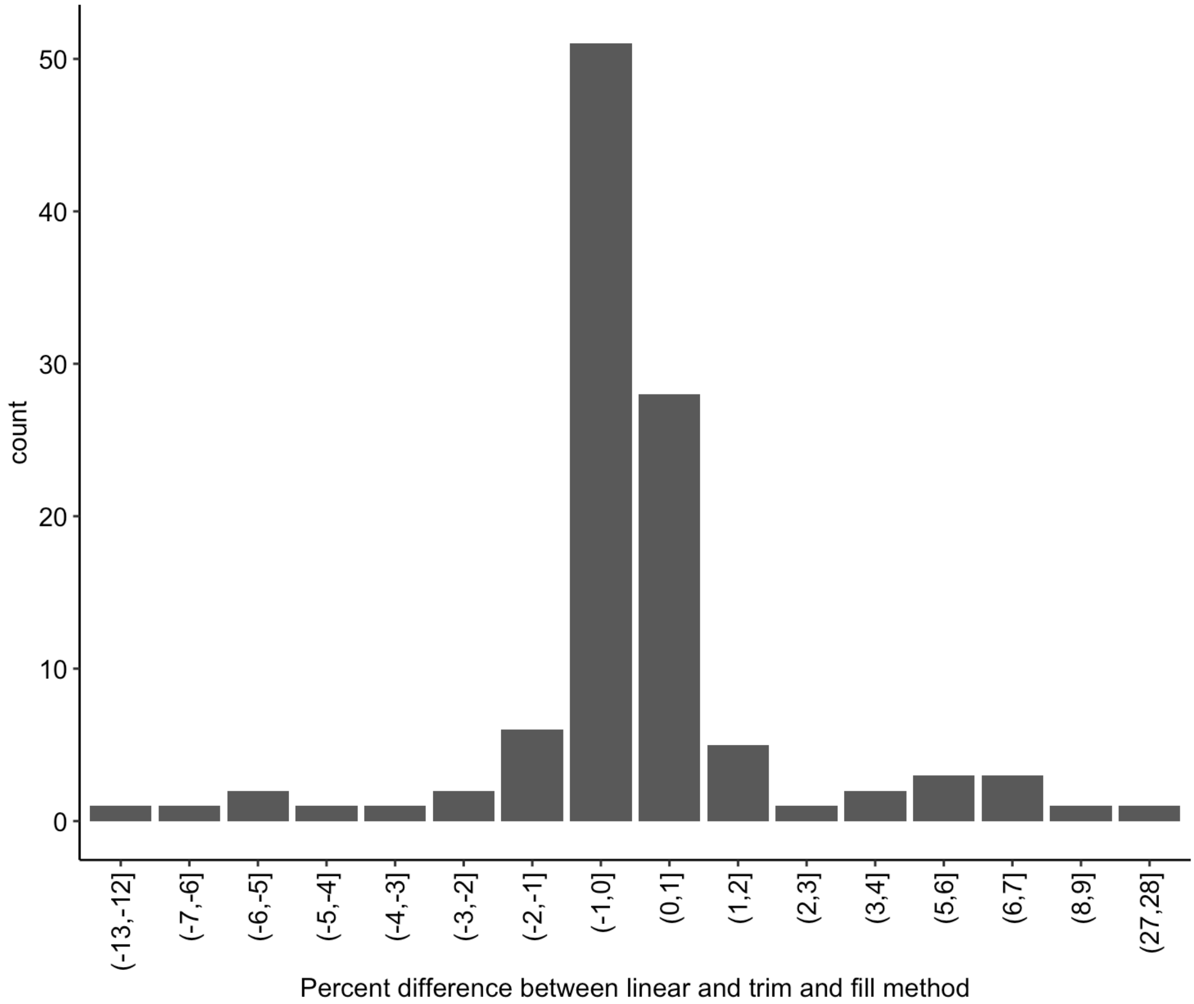

Supplementary Figure 6. Comparison of results (when available) between SOC Stock and SOC concentration for land-use change (a.), Management (b) and Climate change (c) factors.. Only few comparisons were available to compare results between SOC stock and SOC concentration.

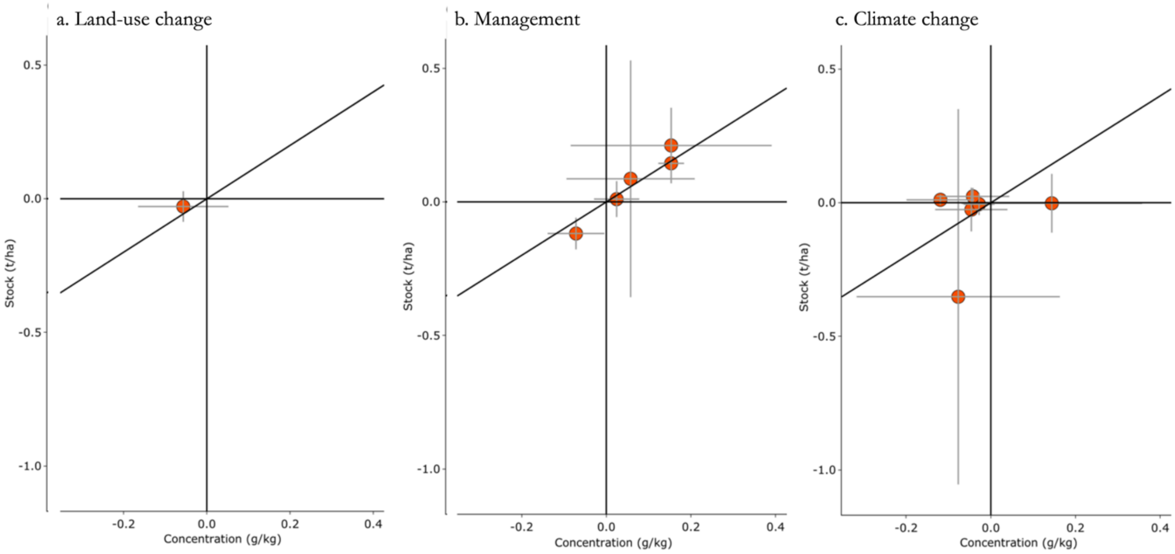

Supplement: Supplementary file 1 — Supplementary Information [file 41467_2023_39338_MOESM1_ESM.pdf]
