# Supplementary material for: A global meta-analysis of soil organic carbon in the Anthropocene
Source: Nat Commun. 2023 Jun 22;14:3700. doi: 10.1038/s41467-023-39338-z (PMC10287672; doi:10.1038/s41467-023-39338-z)
Supplement: Supplementary file 3 — Description of Additional Supplementary Files [file 41467_2023_39338_MOESM3_ESM.pdf]

Description of Additional Supplementary Files:

Supplementary Dataset 1. Percentage change in soil organic carbon (SOC) resulting from land-use change, land management, and climate change, according to soil depth. An interactive version of the plot is available at https://rpubs.com/dbeillouin/Supl_soil_depth

Supplementary Dataset 2. Percentage change in soil organic carbon (SOC) resulting from land-use change, land management, and climate change detailed according to the type of metric used to quantify soil Carbon. Warning; these results are obtained with a nonsystematic review of the literature. An interactive version of the plot is available at: https://rpubs.com/dbeillouin/Suppl_All_effects

Supplementary Dataset 3. Percentage change in soil organic carbon (SOC) resulting from a combination of intervention related to land-use change, land management, and climate Damien Beillouin Agronomy CIRAD, Hortys Lab damien.beillouin@cirad.fr change. An interactive version of the plot is available at: https://rpubs.com/dbeillouin/Combi_practices
